# Various surgical techniques to create an aesthetic appearance at the donor site of anterolateral thigh free flaps based on the oblique branch: Twenty-one clinical case reports

**DOI:** 10.1097/MD.0000000000009885

**Published:** 2018-02-16

**Authors:** Chengliang Deng, Hai Li, Zairong Wei, Wenhu Jin, Kaiyu Nie, Shujun Li, Bihua Wu, Dali Wang

**Affiliations:** Department of Plastic Surgery, Affiliated Hospital of Zunyi Medical College, Zunyi, Guizhou, People's Republic of China.

**Keywords:** aesthetic appearance, anterolateral thigh flap, donor site, oblique branch, surgical techniques

## Abstract

**Rationale:**

Reducing the morbidity associated with anterolateral thigh (ALT) donor sites by performing aesthetic restoration has become a popular research topic. Various surgical techniques have been developed allowing for direct closure of the donor site. However, closure techniques for ALT donor sites based on the oblique branch have not been systematically reported.

**Patient concerns:**

Data from 21 patients (18 males, 3 females) undergoing operative reconstruction with an ALT free flap between January 2016 and December 2016. The mean age of the participants was 42 years (range, 18–60 years).

**Diagnoses:**

The soft-tissue defects resulted from a traffic accident in 9 patients, a fall injury in 7 patients, a machinery injury in 3 patients, an electrical injury in 1 patient, and a burn scar in 1 patient. The wound areas ranged from 6 × 3.5 to 28 cm × 10 cm.

**Interventions:**

Several surgical techniques, including the split skin paddle technique and utilization of an adjacent perforator flap or an ipsilateral groin flap, were utilized to facilitate direct closure of the ALT flap donor site.

**Outcomes:**

Of the 21 patients included in the study, the donor sites were directly sutured in 14 patients (8 of which required a split skin paddle technique). Four patients required an adjacent perforator flap, and 3 patients received an ipsilateral groin flap. The size of the adjacent perforator flaps ranged from 15 × 5 to 17 × 6 cm. The groin flaps ranged from 18 × 6 to 28 × 6 cm. All the flaps had excellent appearance and texture. A linear scar in the donor area was not conspicuous and achieved an aesthetic appearance.

**Lessons:**

The ALT flap donor site based on the oblique branch pedicle can be directly closed without skin grafts through the use of several surgical techniques.

## 1 Introduction

The anterolateral thigh (ALT) flap is one of the most prevalent free flaps used to reconstruct numerous different complicated defects resulting from trauma, tumor resection, and congenital malformations.^[1]^ ALT flaps have many advantages, including good vessel diameter, a long vascular pedicle, and the availability of different tissues with large amounts of skin.^[2,3]^ With better understanding of flap anatomy and the development of flap surgery and microsurgery, the survival rate of ALT free flaps has reached approximately 98%.^[4]^ Consequently, reducing donor site morbidity for aesthetic restoration has become an important area of research.^[5]^

According to the literature, morbidity from direct closure of the ALT donor site is minimal. When direct closure is not possible, a split-thickness skin graft is conventionally used to cover the donor site defect. In this case, morbidity can be high due to scar contraction, sensory disturbance, conspicuous scar, and abnormal pigmentation.^[6]^ Therefore, various surgical techniques have been developed to directly close the donor site without skin grafts, including island V–Y advancement flaps,^[7]^ 2 rectangular advancement flaps,^[8]^ groin flaps,^[9]^ and tissue expansion,^[10]^ to name a few. Each technique has its own indications.^[11,12]^

The oblique branch of the lateral circumflex femoral artery (LCFA) was first described by Wong et al^[13]^ in 2009 and is considered an alternative vascular pedicle to the conventional ALT flap. However, different methods of donor site closure for ALT flaps based on the oblique branch have not been systematically reported. We summarize several ways to close the donor site through reporting clinical cases of various ALT flaps based on the oblique branch, such as the split skin paddle technique, introducing adjacent perforator flaps, and ipsilateral groin flaps. To the best of our knowledge, this is the first report in the published literature that systematically describes closure techniques for the donor site of ALT flaps based on the oblique branch.

## 2 Patients and methods

Data from 21 patients (18 males, 3 females) undergoing operative reconstruction with an ALT free flap between January 2016 and December 2016 for soft tissue defects on 1 of the 4 limbs were retrospectively analyzed. All participants provided written informed consent to participate in this study. The mean age of the participants was 42 years (range, 18–60 years). The soft-tissue defects resulted from a traffic accident in 9 patients, a fall injury in 7 patients, a machinery injury in 3 patients, an electrical injury in 1 patient, and a burn scar in 1 patient. The wound areas ranged from 6 × 3.5 to 28 cm × 10 cm; the ALT flap sizes ranged from 8 × 5 to 31 cm × 12 cm.

### 2.1 Surgical technique

**Figure 1:**
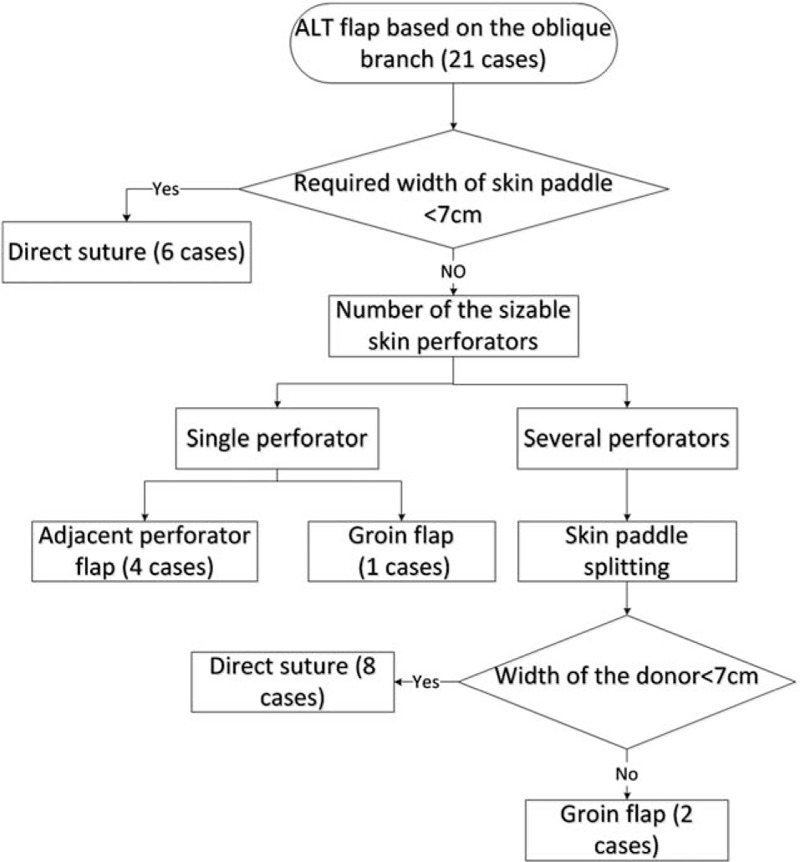
Algorithm for several surgical techniques to facilitate primary closure of the donor site and the number of patients whose donor sites were closed by each method.

Each operation was divided into 2 processes. First, the ALT flap was harvested for the soft tissue reconstruction, with the oblique branch of the LCFA serving as the pedicle. Briefly, a straight line connecting the anterior superior iliac spine and the superior margin of the patella and a circle with a 3-cm radius centered at the midpoint of this line were drawn. According to the Yu ABC system, 1 to 3 cutaneous perforators were identified by handheld Doppler examination and were named perforators A, B, and C from proximal to distal along this line.^[14]^ A medial skin incision was then performed and the flap was elevated. All available perforators were routinely unroofed and traced. Various types of ALT flaps based on the oblique branch vascular pedicle were then harvested according to the wound situation. The resultant defect located at the upper-to-middle third of the thigh was defined by a piece of template. When the width of the required flap was <7 cm, the donor site was directly sutured. When the width was >7 cm and the oblique branch issued several skin perforators, a split skin paddle technique was utilized and the donor site was directly sutured or received an ipsilateral groin flap. When the width was >7 cm and the oblique branch only issued 1 skin perforator, an adjacent perforator flap or an ipsilateral groin flap was utilized (Fig. 1).

### 2.2 Split skin paddle technique

The purpose of skin paddle splitting was to reduce the effective width of the donor site and achieve a donor site wound width of <7 cm. When the width was >7 cm and the oblique branch issued several sizable skin perforators, the donor area was measured and then divided into several separate skin paddles nourished by the same source vessel according to the number of the sizable skin perforators. The wide defect could then be covered by these narrower flaps based on the oblique branch vascular pedicle, while the donor defect could then be directly sutured when it was <7 cm in width.

### 2.3 Adjacent perforator flap

When the width of the required flap was >7 cm and the oblique branch only issued 1 skin perforator, a single paddle ALT flap was harvested. The resultant donor site defect could be repaired by using an adjacent perforator flap originating from the descending branch of the LCFA. The original medial skin incision located on the thigh was appropriately elongated and the flap was elevated. The sizable cutaneous perforators were then identified through subfascial dissection. Subsequently, a skin paddle based on a distal cutaneous perforator originating from the descending branch of the LCFA was raised and advanced into the central portion of the donor site. The second donor area was directly sutured without difficulties.

### 2.4 Ipsilateral groin flap

When the width of the required flap was >7 cm, neither the split skin paddle nor adjacent perforator flap technique was enough to ensure sufficient coverage, and an ipsilateral groin flap technique was utilized. The flap size was determined based on the resultant defect of the donor site and was generally 1 to 2 cm longer than the donor area with a width <8 cm. The vascular pedicle of the groin flap was the superficial circumflex iliac artery (SCIA). The rotation point of the flap was located at the midpoint of the groin ligament. A medial skin incision of the flap was performed. The origin of the SCIA was identified and guarded through the course of the femoral artery, and the deep branch of the circumflex iliac artery was ligated. Subsequently, a skin paddle based on the SCIA and accompanying veins could be raised and rotated into the central portion of the donor site. The second donor area was directly sutured without difficulties. The purpose of introducing new skin flaps into the donor site was to reduce the effective width of the donor site and to facilitate direct closure.

### 2.5 Postoperative details

After surgery, all patients were treated with antibiotics, antivasospastic agents, and anticoagulant therapies. Papaverine hydzochloride injection was the most important antivasospastic agent and the used regimens were intramuscular injections, once 30 mg, Q8 hours, continuous use for 7 days. Anticoagulant therapy was usage of low-molecular-weight heparin and the used regimens were subcutaneous injections, once 4000 U, Q12 hours, and the duration was 7 days. Capillary filling time, graft color, and temperature of the skin flap were recorded. The arterial infusion and venous drainage in the skin flap were evaluated. Within 2 weeks, the sutures were removed, and patients were allowed to ambulate.

## 3 Results

Of the 21 study participants, the donor sites were directly sutured in 14 patients, with 8 patients needing a split skin paddle technique. Adjacent perforator flaps were introduced in 4 patients, and 3 patients received an ipsilateral groin flap (Table 1). Postoperative wound dehiscence at the donor site was not encountered in any of the patients. All patients got healed by primary intention. Of the 21 ALT flaps, a single paddle of 1 double-paddled flap suffered vascular crisis and was eventually corrected through a second remedial surgery. The size of the adjacent perforator flaps ranged from 15 × 5 to 17 × 6 cm, and the groin flaps ranged from 18 × 6 to 28 × 6 cm. Follow-up for all patients ranged from 1 to 12 months, with an average follow-up period of 7 months. All the flaps had excellent appearance and texture at follow-up. A linear scar was not prominent in the donor area. None of the patients experienced residual local pain, sensory disturbance, or abnormal pigmentation. The stretch function of the knee was not reduced in any of the patients.

**Table 1:**
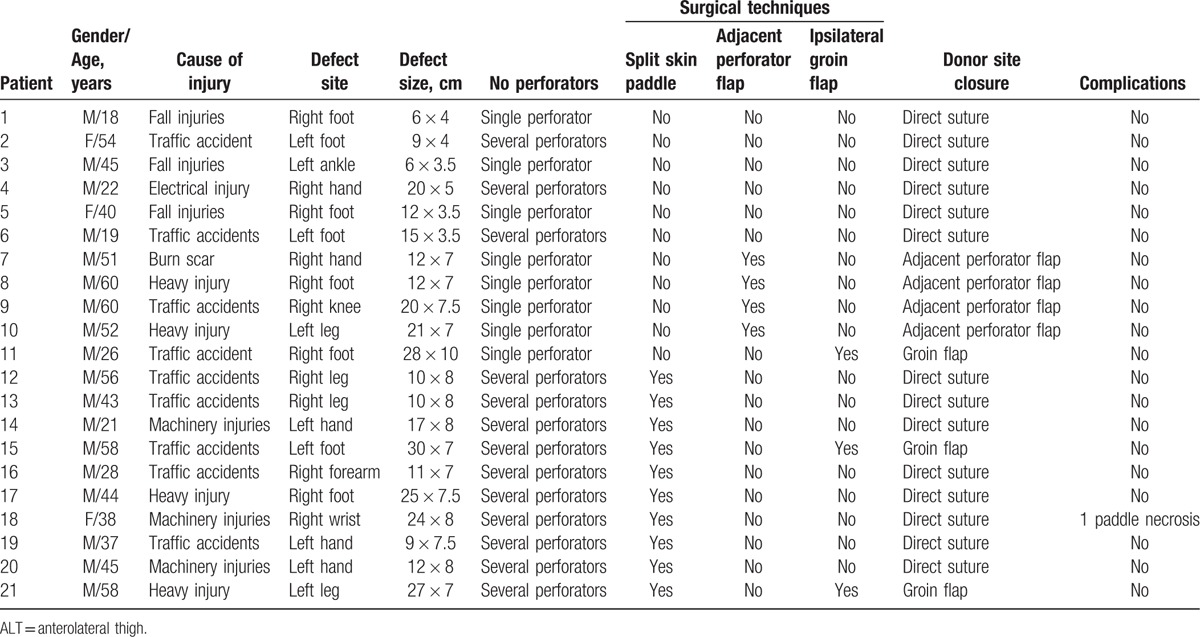
Summary of 21 patients underwent ALT free flaps surgery.

## 4 Classical cases

### 4.1 Case 13

**Figure 2:**
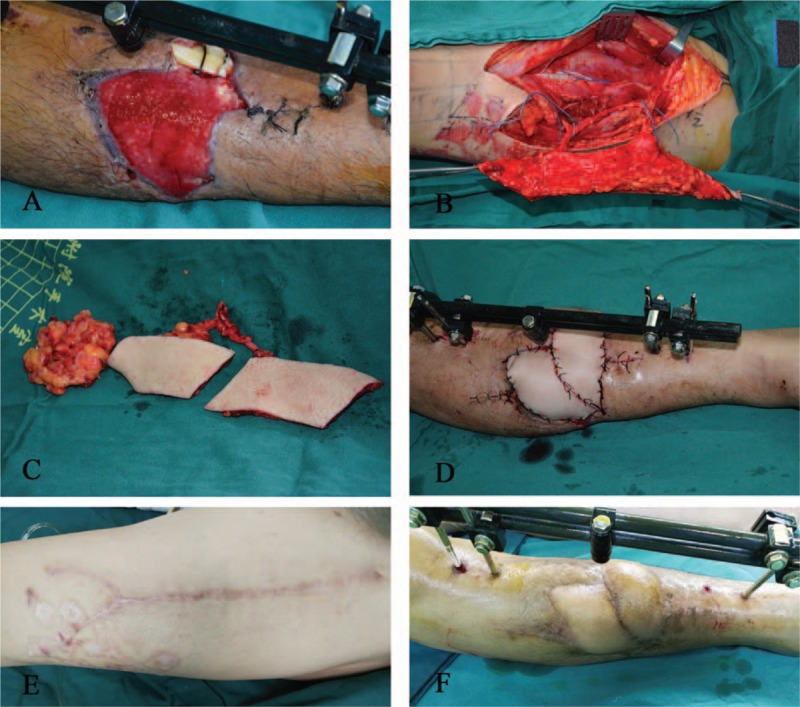
The split skin paddle technique was utilized. (A) Preoperative view: the wound located on the right leg. (B) Elevation of the ALT flap based on the oblique branch. (C) The split skin paddle technique. (D) Early postoperative view of the flap placement. (E) The appearance of the donor site after direct suturing 5 months postoperatively. (F) The appearance of the flap 5 months postoperatively. ALT = anterolateral thigh.

A 43-year-old man was admitted to the Department of Plastic Surgery for suspected tibial osteomyelitis resulting from a skin defect and tibial fracture that remained exposed 1 year after external fixation. Three days later, a flap surgery was performed under general anesthesia. The wound was located on the right leg and was irregular, with the widest point measuring 8 cm and the longest measuring 10 cm. A contralateral ALT flap was harvested. The oblique branches were found intraoperatively to issue 2 skin perforators, and the split skin paddle technique was utilized. The flap was divided into 2 paddles, with the widest measuring 5 cm. The double paddles were used to cover the skin defect. The donor area was directly sutured. The flaps survived, and the wounds got healed by primary intention. The flaps had an excellent appearance and texture at a 5-month follow-up. A linear scar was visible at the donor site; however, there was no local pain or hyperaphia (Fig. 2).

### 4.2 Case 7

**Figure 3:**
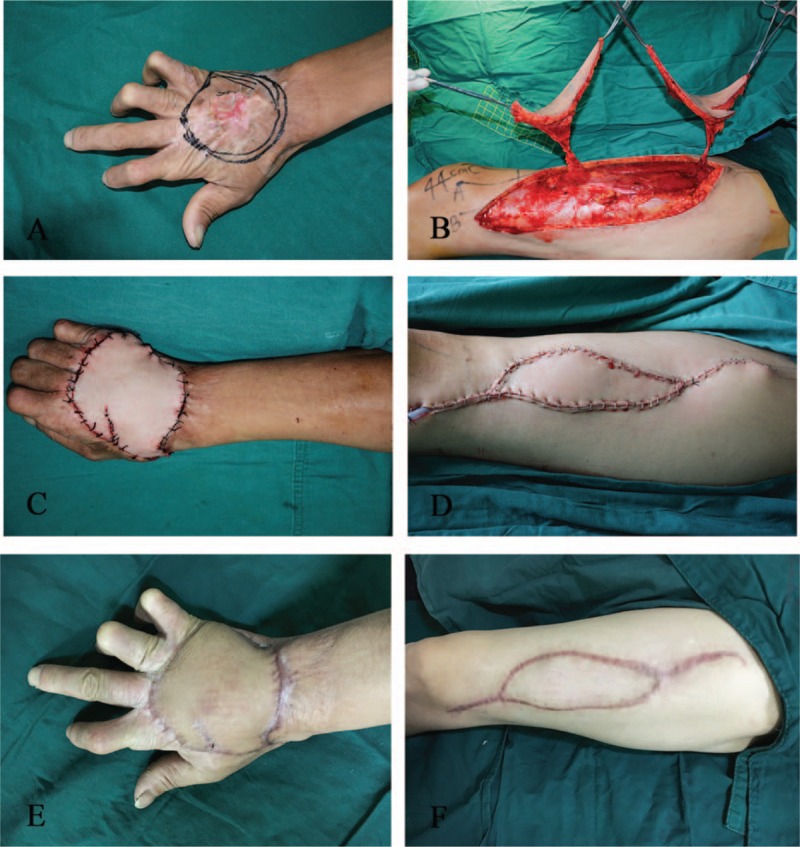
An adjacent perforator flap. (A) Preoperative view: the ulcerated scar located on the back of the right hand. (B) Elevation of the 2 ALT flaps. (C) Early postoperative view of the flap placement on the right hand. (D) Early postoperative view of the flap placement at the donor site. (E) The appearance of the flap 7 months postoperatively. (F) The appearance of the donor site 7 months postoperatively. ALT = anterolateral thigh.

A 51-year-old man was admitted to the Department of Plastic Surgery for an ulcerated scar caused by a burn 40 years prior located on his right hand. Five days later, a flap surgery was performed under general anesthesia. Intraoperative frozen pathology of the ulcer tissue was performed, and results returned as squamous cell carcinoma. Therefore, a radical resection of the ulcer tissue was performed, resulting in a wound measuring 12 × 7 cm. A contralateral ALT flap was designed. The patient's oblique branches were found intraoperatively to only issue 1 skin perforator, so a single paddle was harvested. The free flap was used to cover the skin defect, leaving a resultant defect located in the donor area. An adjacent perforator flap originating from the descending branch of the LCFA was elevated and advanced into the central portion of the donor site. The second donor area was directly sutured. The flaps survived, and the wounds healed by primary intention. There was no local pain or sensory disturbance in the donor site (Fig. 3).

### 4.3 Case 21

**Figure 4:**
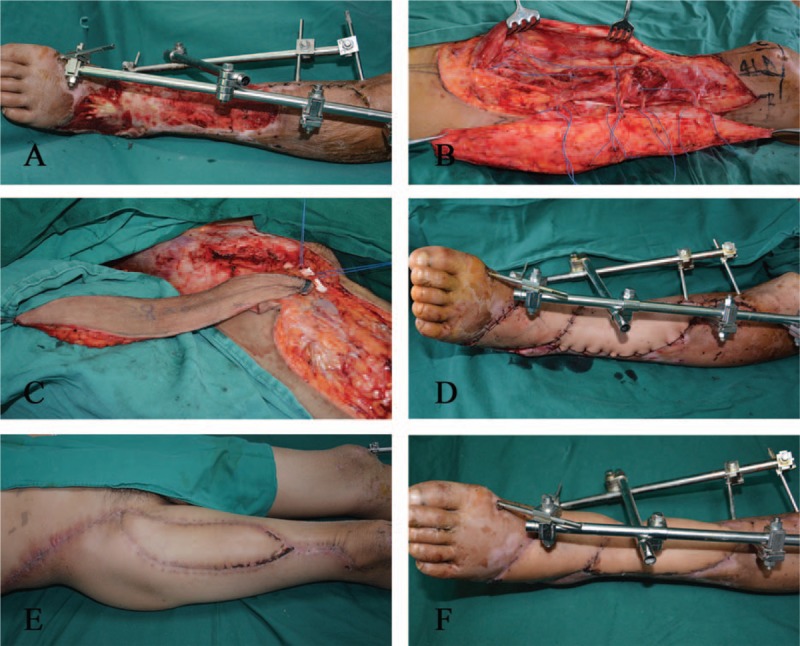
An ipsilateral groin flap. (A) Preoperative view: the wound located on the left leg. (B) Elevation of the ALT flap based on the oblique branch. (C) Elevation of the ipsilateral groin flap. (D) Early postoperative view of the flap placement. (E) The appearance of the donor site 3 months postoperatively. (F) The appearance of the flap 3 months postoperatively. ALT = anterolateral thigh.

A 58-year-old man was admitted to the Department of Plastic Surgery for a skin defect on the left leg caused by a heavy injury 5 hours prior. One week later, a flap surgery was performed under general anesthesia. The wound measured 27 × 7 cm, and a contralateral ALT flap was designed. The oblique branches were found intraoperatively to issue several skin perforators, and the split skin paddle technique was utilized. The harvested double-paddled flap was used to cover the skin defect. An ipsilateral groin flap was used to repair the resultant defect located in the donor area. The 2 flaps survived, and the wounds healed by primary intention. At a 3-month follow-up, the flaps had excellent appearance and texture. The donor site achieved an aesthetic appearance (Fig. 4).

## 5 Discussion

It has been reported that up to 20% of ALT flap donor sites cannot be closed by primary intention. This significant percentage of patients can still endure donor-site morbidities, mostly resulting in the need for additional skin grafting.^[6,15]^ However, with the development of reconstructive techniques, better reconstruction and restoration can be achieved not only at the recipient site but also at the donor site.^[12]^ In our study, a variety of surgical techniques were used to accomplish direct closure of the donor site, resulting in a cosmetic and functional outcome. To the best of our knowledge, this is the first article to systematically elaborate on closing techniques for the donor area of ALT flaps based on an oblique branch pedicle.

Generally, the ratio of the flap width to thigh circumference is the most important factor that decides whether or not the ALT donor site can be closed directly. A flap width <16% of the thigh circumference or the defect width <8 cm is generally amenable to direct primary suturing.^[16,17]^ In our clinical practice, if the width of the ALT flap is <7 cm, the donor area can be easily closed in most adults. Therefore, in designing the donor flaps, we reduce the width of the required flap mainly through the skin paddle splitting technique and paddle recombination to achieve a maximum width of the donor site of ≤7 cm. The split skin paddle ALT flap is a modification of the typical ALT flap that expands the clinical applications of ALT flaps for reconstructing very large skin tissue defects while maintaining the ability to achieve primary closure of the donor site.^[18]^ The skin paddle splitting technique possesses 2 merits: first, a better aesthetic appearance at the donor site can be achieved and second, the skin paddles can be inset with a greater degree of freedom, improving the cosmetic appearance of the recipient site.^[18]^ In our study, if the oblique branch issued ≥2 skin perforators, we tried to use this technique. Of the 21 patients, a skin paddle splitting technique was performed in 10 patients. Most of the ALT polyflaps survived except for a single paddle of 1 double-paddled flap that suffered vascular crisis. Vascular crisis may be caused by vascular torsion; 2 skin paddles increases the risk of kinking or twisting of the vascular pedicle. Attention and maximal care to prevent this complication is needed when ALT polyflap insets are used.^[18]^ However, when the oblique branch only issues a single skin perforator or when the required width of the donor site cannot effectively be reduced using a skin paddle splitting technique, then the introduction of an adjacent flap may need to be considered.

The location of the donor site is significantly different when comparing ALT flaps based on the oblique branch and those based on the descending branch vascular pedicle.^[13]^ In general, the donor area of the descending branch pedicle is located in the middle-to-distal third of the thigh; however, the donor area in ALT flaps made from the oblique branch is located in the upper-to-mid third. The upper-to-mid third of the thigh is a very convenient location to introduce a variety of local flaps, such as a local perforator flap based on the descending branch or an ipsilateral groin flap. If the oblique branch only issues 1 skin perforator, then there are guaranteed to be skin perforators deriving from the descending branch in the middle-to-distal third of the thigh; under these circumstance, a middle-to-distal perforator flap and groin flap can be introduced.^[12]^ If the oblique branch issues multiple perforators and most of the skin on the ALT is used as a flap, then a local perforator flap is less likely to be an option, and a groin flap may be the only option to create a local flap.

An adjacent perforator flap based on the descending branch is generally located in the middle-to-distal third of the thigh, where a traditional ALT flap is harvested.^[1]^ The foremost advantages of this perforator flap are that it provides the color, texture, thickness, and structures that maximally resemble those of the ALT donor site based on the oblique branch.^[19]^ In our study, the donor area obtained a better cosmetic appearance through introducing an adjacent perforator flap based on the descending branch, as previously reported.^[19]^ However, the possibility of vascular crisis from kinking or twisting of the vascular pedicle should be considered, especially in flaps that are rotated up to 180°.^[19]^ In our experience, the vascular pedicle does not carry much fascia and a portion of the muscles are routinely removed at the point where the skin perforator pierces the muscle, so that the vascular pedicle retains sufficient space.

The groin area is commonly used as a skin grafting donor area. The groin flap was first reported by McGregor and Jackson in 1972.^[20]^ It possesses various merits, including providing a flap with the color and texture that resemble the thigh donor site, a completely hidden and unobtrusive scar, and a flap that requires minimal trimming.^[9,21,22]^ One notable disadvantage of using a groin flap as a local flap is the limited vascular pedicle length.^[9,23]^ Consequently, repairing thigh skin defects by using a groin flap is limited to defects positioned on the upper-to-mid third of the thigh.^[23]^ Unlike the descending branch, the donor area of the oblique branch vascular pedicle is located at the upper-to-mid third of the thigh. Therefore, the resultant defect may be the best indication for a groin flap. In our study, 3 groin flaps were used and survived, and the donor site of the ALT flap based on the oblique branch had a cosmetically appealing outcome.

A tissue expansion technique can also be used to improve chance of primary closure in the ALT flap donor area, but is time consuming.^[10]^ Advancement of 2 rectangular flaps is used to achieve direct primary closure of the donor site without skin grafting, but only to limited repairable defect areas.^[8]^ Additionally, if a huge defect is encountered, a free flap may be need.^[23,24]^ All in all, avoiding needing a skin graft for the donor area and minimizing donor-site morbidities are areas of concern in plastic surgery.

Our study was not without limitations. The information presented in this study is restricted by its lower sample size and shorter follow-up time. In addition, sophisticated surgical skills are required because sometimes several perforator flaps and vascular anastomosis technique are performed in a short time. In a word, the ALT flap donor site based on the oblique branch pedicle can be directly closed without skin grafts through the use of several surgical techniques.

## Acknowledgment

This manuscript has been thoroughly edited by a native English speaker from an editing company.
